# Bioactive Compounds from *Ephedra fragilis*: Extraction Optimization, Chemical Characterization, Antioxidant and AntiGlycation Activities

**DOI:** 10.3390/molecules26195998

**Published:** 2021-10-02

**Authors:** Ismail Guenaou, Imane Nait Irahal, Ahmed Errami, Fatima Azzahra Lahlou, Fouzia Hmimid, Noureddine Bourhim

**Affiliations:** 1 Laboratoire Santé Et Environnement, Faculté Des Sciences Ain Chock, Université Hassan II de Casablanca, B.P 5366 Maarif, Casablanca 20000, Morocco; ismail.guenaou@gmail.com (I.G.); imanenaitirahal@gmail.com (I.N.I.); fzlahlou@um6ss.ma (F.A.L.); fouzia.hmimid@gmail.com (F.H.); 2 Laboratoire d’Ingénierie Des Procédés Et D’Environnement, École Supérieure De Technologie, Université Hassan II de Casablanca, B.P. 8012, Casablanca 20000, Morocco; a_errami@yahoo.fr; 3 Laboratoire National De Référence, Université Mohammed VI Des Sciences De La Santé Faculté De Médecine, B.P 82 403, Casablanca 20000, Morocco; 4 Équipe de Biotechnologie, Environnement Et Santé, Faculté Des Sciences El Jadida, Université Chouaïb Doukkali, B.P 299, El Jadida 24000, Morocco

**Keywords:** *Ephedra fragilis*, response surface methodology, Box-Behnken design, bioactive compounds, RP-HPLC, antioxidant and antiglycation activities

## Abstract

Response surface methodology (RSM) with a Box–Behnken design (BBD) was used to optimize the extraction of bioactive compounds from *Ephedra fragilis*. The results suggested that extraction with 61.93% ethanol at 44.43 °C for 15.84 h was the best solution for this combination of variables. The crude ethanol extract (CEE) obtained under optimum extraction conditions was sequentially fractionated with solvents of increasing polarity. The content of total phenolic (TP) and total flavonoid (TF) as well as the antioxidant and antiglycation activities were measured. The phytochemical fingerprint profile of the fraction with the highest activity was characterized by using RP-HPLC. The ethyl acetate fraction (EAF) had the highest TP and TF contents and exhibited the most potent antioxidant and antiglycation activities. The Pearson correlation analysis results showed that TP and TF contents were highly significantly correlated with the antioxidant and antiglycation activities. Totally, six compounds were identified in the EAF of *E. fragilis*, including four phenolic acids and two flavonoids. Additionally, molecular docking analysis also showed the possible connection between identified bioactive compounds and their mechanisms of action. Our results suggest new evidence on the antioxidant and antiglycation activities of *E. fragilis* bioactive compounds that may be applied in the treatment and prevention of aging and glycation-associated complications.

## 1. Introduction

The continuous exposure to aggressors from various sources may lead to a rise in free radicals production in the human body, exceeding its capacity to regulate them, and, over time, contributes to the development of several oxidative stress-associated diseases including ageing and diabetes [1]. Hence, antioxidants supplementation can help to maintain an optimal biological system by removing excessive concentrations of free radicals [2]. It has been demonstrated that free radicals participate in the glycation process. Glycation, a spontaneous nonenzymatic reaction between available amino groups of amino acid residues in proteins and reducing sugars, occurs to a higher extent under aging and hyperglycemia, resulting in advanced glycation end products (AGEs) production and accumulation [3]. Aside from it interfering with proteins and altering their functionality, AGEs can also engage with the receptor for AGEs (RAGE), a 45 KDa multi-ligand-cell surface receptor belonging to the immunoglobulin superfamily [4], and activate several downstream intracellular signaling pathways accompanied by a rise in free radicals production that contribute towards pathologic complications related to diabetes [5]. 

It is well established that antioxidants and radical scavenger molecules are good protectors against these processes [6]. The use of medicinal plants in preventing and counterbalancing diseases associated to oxidative stress is an old medical tradition. Recently, many studies have demonstrated that secondary metabolites such as tannins, phenolic acids, and flavonoids with dual antioxidant and antiglycation potential are more effective in treating diabetes mellitus [7]. Therefore, identifying new sources of phytochemicals that effectively scavenge free radicals and reduce non-enzymatic glycation is a great interest. 

*Ephedra fragilis* is a member in the Ephedra genus (Ephedracea family) that contains more than 60 species growing in desert and semiarid conditions in both hemispheres across six continents [8]. For more than 5000 years, many species in the Ephedra genus have been commonly used in traditional Chinese medicine (TCM) for the treatment of several diseases; there have been several studies reporting their multiple health benefits such as anti-inflammatory [9], anti-invasive, anti-angiogenic, [10], antimicrobial, antiproliferative, pro-apoptotic [11], neuroprotective [12], hepatoprotective, and anti-oxidant properties [13]. Flavonoids, alkaloids, phenolic acids, and other compounds in Ephedra plants have been considered as the main phytochemical components for these pharmacological properties [14]. In the pharmaceutical industry, reverse-phase high-performance liquid chromatography (RP-HPLC) is widely employed as an analytical method to detect and identify chemical compounds based on their different hydrophobic properties, although few studies are described in the literature for chemical analysis of Ephedra species.

The extraction process of these bioactive compounds from different plants sources is the first important step involved in their qualitative and quantitative analysis [15]. Different factors viz. extraction method, solvent type and concentration, temperature, time, and others can significantly influence the composition and extraction rate of these compounds [16]. Therefore, optimization of the extraction processes is required to yield a high content of plant active compounds. Originally developed in the 1950s by Box and Wilson, response surface methodology (RSM) is nowadays the most commonly used tool to perform, improve, and optimize such processes in which independent factors have a combined effect on the desired response. One of the most frequently selected designs in RSM by researchers is the Box–Behnken design (BBD) because it needs a limited trial and, therefore, represents an alternative that avoids long-time experiments and decreases expenses [17,18,19]. 

Up to date, no studies are available in the literature regarding the extraction optimization of *E. fragilis* bioactive compounds. Therefore, this study aimed to optimize the extraction of total phenolic (TP) and flavonoid (TF) contents from *E. fragilis* by applying a BBD. The crude ethanol extract (CEE) obtained under optimum extraction conditions was sequentially fractionated with solvents of increasing polarity, and their antioxidant and antiglycation activities were investigated using different in vitro tests. Finally, we performed in silico analysis to further understand the mechanisms by which bioactive compounds in EAF, as identified by reverse-phase high-performance liquid chromatography (RP-HPLC), bind to BSA and RAGE as target proteins. 

## 2. Results and Discussion

### 2.1. Fitting the Models

RSM with a BBD was applied to investigate the effect of ethanol concentration (%, $X_{1}$), temperature (°C, $X_{2}$), and time (h, $X_{3}$) on the extraction yield of TP and TF from *E. fragilis*. The results of 15 trials after the BBD are given in Table 1. Analysis of variance (ANOVA) (Table 2) indicates that the models were significant as evidenced by F and *p*-values. The coefficient of multiple determinations (R^2^) of the models were 0.9935 and 0.9939 for TP and TF, respectively, suggesting that only 0.65 and 0.61% of the total variations are not explained by the models. A comparable value of adjusted R^2^ to R^2^ represents an excellent statistical model. As given in Table 2, the adjusted R^2^ (0.9818 and 0.983 for TP and TF contents, respectively) is close to R^2^, which means that the insignificant terms were not included in the models. Moreover, predicted R^2^ (0.9012 and 0.9338 for TP and TF contents, respectively) is in reasonable agreement with adjusted R^2^ and confirms that the models are highly significant. The ‘‘fitness’’ of the models was also confirmed using lack of fit test. The insignificant *p*-value for lack of fit (*p* > 0.05) for two responses indicates the suitability of models for accurate prediction of the variation in the results [20]. A good precision is described as a signal to noise ratio greater than 4, which is considered desirable [21]. The values of adequate precision are 29.4772 and 28.729 for TP and TF contents, respectively, demonstrating an adequate signal. Simultaneously, the smaller values of coefficient of variation (C.V.%) (1.52 and 0.4246 % for TP and TF contents, respectively) indicate better precision and reliability of experimental values. For each response factor, the influence of the extraction factors $X_{1}$ (ethanol concentration), $X_{2}$ (extraction temperature), and $X_{3}$ (extraction time) was carefully examined (Table 2). The significance of each coefficient was tested using *F* and *p*-values, considering that a greater *F*-value and a smaller *p*-value always led to more significant correspondence between various independent variables [22]. Collectively, these results indicated that the models were reproducible and were suitable for optimization.

### 2.2. Effects of Extraction Variables on TP Content

As given in Table 2, the ANOVA results showed significant linear ($X_{1}$ and $X_{2}$), quadratic ($X_{1}^{2}$, $X_{2}^{2}$ and $X_{3}^{2}$), and interactive ($X_{1}X_{2}$ and $X_{2}X_{3}$) effects on TP content. Among these, TP content is mainly dependent on $X_{2}$, $X_{1}^{2}$, $X_{2}^{2}$, $X_{3}^{2}$, and $X_{2}X_{3}$ at *p* < 0.001 followed by $X_{1}$ and $X_{1}X_{2}$ at *p* < 0.01. The following second order polynomial equation could be used to express the relationship between TP content and variables:

(1) $$\mathrm{YTP}=-8.52+0.3328\;X_{1}+0.4447\;X_{2}+0.4143\;X_{3}-0.002164\;X_{1}^{2}-0.004810\;X_{2}^{2}\;-0.02349\;X_{3}^{2}-0.001614\;X_{1}X_{2}+0.000694\;X_{1}X_{3}+0.006270\;X_{2}X_{3}$$

The value of lack of fit was non-significant (*F*-value = 11.02, *p*-value = 0.0843), indicating that the model is well-fitted with good prediction (R^2^ = 0.9935; Adj R^2^ = 0.9818) (Table 2).

**Figure 1:**
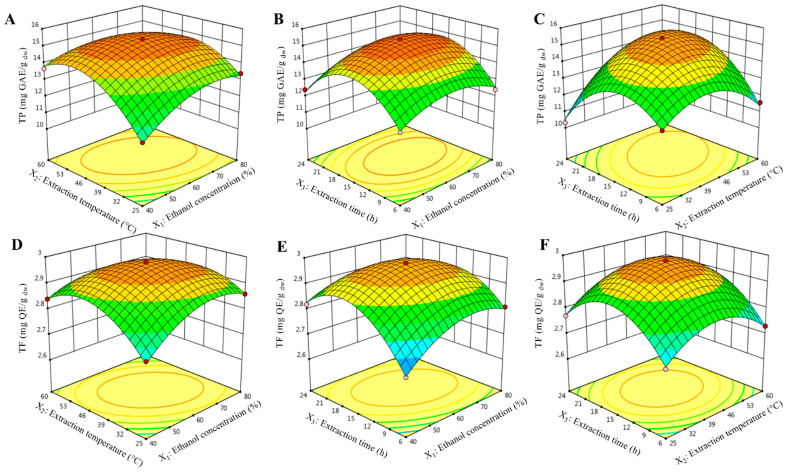
3D plot for interactions between independent variables on extraction of total phenolic (TP, in mg GAE/g dw, **A**–**C**) and flavonoid content (TP, in mg QE/g dw, **D**–**F**).

The interactions between ethanol concentration and extraction temperature ($X_{1}X_{2}$) produce a highly significant (*p* < 0.01) effect on TP content (Table 2). As ethanol concentration ($X_{1}$) and extraction temperature ($X_{2}$) increase in the range of 40–61.80% and 25–44.30 °C, respectively, the TP content increases rapidly. However, beyond 61.80% and 44.30 °C, TP content decreases slightly (Figure 1A). However, the interaction of the extraction temperature and extraction time ($X_{2}X_{3}$) showed a high significant (*p* < 0.001) effect on TP content (Table 2). As shown in Figure 1B, TP content slightly improved with increasing extraction temperature ($X_{2}$) and extraction time ($X_{3}$) up to 44.37 °C and 15.77 h, respectively, but diminished slowly thereafter. 

These effects could be due to the fact that phenolic compounds are polar molecules that naturally occur with glucosides, which make them more water-soluble [23]. As the extraction of phenolic compounds is strongly dependent on the solvent polarity, a water–alcohol mixture is more effective in their extraction than alcohol alone [20]. In regard to the “like-dissolves-like” principle, a decrease in the ethanol concentration leads to an increase in the polarity of solvent, which helps dissolving TP [15]. Nevertheless, a high ethanol concentration can influence the extraction rate by preventing the dissolution of phenolic compounds. Likewise, an increase in the extraction temperature enhanced the recovery of target phenolic compounds by softening of tissues, weakened the cell wall integrity, enhanced mass transfer and penetration of solvent into the plant matrix, and increased both solubility and diffusion rate; however, temperatures too high for an extended extraction time may increase the chances of their degradation [23]. Furthermore, a long extraction period has been found to potentially extend oxygen and light exposure, which ultimately enhances the risk of free-radicals formation that can be scavenged by phenolic compounds [23]. Therefore, an extended extraction time was not helpful to maximize the extraction yield [24].

### 2.3. Effects of Extraction Variables on TF Content

As evident from Table 2, the linear effects of $X_{1}$ and $X_{3}$; quadratic effects of $X_{1}^{2}$, $X_{2}^{2}$ and $X_{3}^{2}$; and the interaction effect of $X_{1}X_{2}$ and $X_{1}X_{3}$ demonstrated significant effects on TF content. Among all significant factors, TF is mainly dependent on $X_{3}$, $X_{1}^{2}$, $X_{2}^{2}$, $X_{3}^{2}$, and $X_{1}X_{2}$ at *p* < 0.001 followed by $X_{1}$ and $X_{1}X_{3}$ at *p* < 0.01. The fitted second order polynomial of TF content is as follows:

(2) $$Y_{\mathrm{TF}}=+0.8401+0.03104\;X_{1}+0.03279\;X_{2}+0.05933\;X_{3}-0.000182\;X_{1}^{2}-0.000303\;X_{2}^{2}\;-0.001517\;X_{3}^{2}-0.000121\;X_{1}X_{2}-0.000208\;X_{1}X_{3}+0.000048\;X_{2}X_{3}$$

The non-significant value of lack of fit (*F*-value = 1.13; *p*-value = 0.5024) suggested that the proposed model fitted to the spatial influence of the variables to the response with good prediction (R^2^ = 0.9939; Adj R^2^ = 0.9830) (Table 2). 

Various 3D response surface graphs were generated for TF content and shown in Figure 1D–F. The interaction effect of ethanol concentration and extraction temperature ($X_{1}X_{2}$) showed a significant (*p* < 0.001) effect on TF content. From Figure 1D, TF content increased at first and then decreased quickly with the rise of the two parameters, and a maximum TF content was achieved when ethanol concentration ($X_{1}$) and extraction temperature ($X_{2}$) were 61.89% and 44.23 °C, respectively. This phenomenon is similar to TP, which might also be attributed to the fact that a rise in the extraction temperature, the solubility, extraction rate, and diffusion rate increases, which ultimately helps TF to dissolve in solvent [25]. 

Similarly, the interaction between ethanol concentration and extraction time ($X_{1}X_{3}$) showed a similar correlation (Figure 1F). The extraction yield of TF gradually increased with increasing of both ethanol concentration ($X_{1}$) and extraction time ($X_{3}$). Near the midpoint of the response plot (61.89% and 15.81 h for $X_{1}$ and $X_{3}$, respectively), TF yield reached its highest, but decreased slowly thereafter. This phenomenon is most likely due to Fick’s second law of diffusion principle revealing that the final equilibrium between the solution concentration in the solid matrix and solvent will be attained after a particular duration, leading to deceleration in the extraction yield of target compounds [26].

### 2.4. Validation of Optimized Conditions

The aim of the optimization was to determine the extraction conditions that would provide simultaneously the highest TP and TF contents. Design expert software was used to carry out optimization. The BBD proposed the optimal ethanol concentration, extraction temperature, and time to be 61.93%, 44.43 °C, and 15.84 h, respectively, for the extraction of *E. fragilis* bioactive compounds. At this optimal point, the predicted TP and TF contents were 15.335 mg GAE/g dw and 2.972 mg QE/g dw, respectively (Table 3). A validation of the predictive capacity of the models was performed experimentally under the optimal conditions obtained from RSM. Experiments were carried out in triplicate under the obtained conditions, and the mean TP and TF contents were 14.98 ± 0.29 GAE/g dw and 2.92 ± 0.09 QE/g dw, respectively. The experimental values of investigated responses were comparable and in line with those of predicted values, which confirmed that the models were sufficient to reflect the expected optimization.

### 2.5. Extraction Yield and Phytochemical Analysis

Table 4 showed the extraction yield of *E. fragilis* CEE and its fractions. Our results showed that the extraction yield in different fractions differs significantly from 0.78 to 10.6% (*w*/*w*). As shown in Table 4, the CEE (10.6%) had the highest percentage yield, followed by WF (2.73%), WBF (2.04%), DMF (0.64%), and EAF (0.93%), whereas HF (0.78%) had the lowest percentage yield. 

TP content of *E. fragilis* CEE and its fractions was determined through a regression equation of calibration curve (y = 0.009x − 0.0154; R^2^ = 0.9973) and expressed in milligrams of gallic acid equivalents (GAE) per gram of dried weight (Table 4). Generally, EAF had the highest TP content (32.78 ± 0.49 mg GAE/g DW) followed successively by WBF, DMF, and CEE (25.02 ± 1.01, 19.21 ± 0.22 and 14.98 ± 0.29 mg GAE/g DW), whereas, the WF and HF presented the lowest contents (10.47 ± 0.71 and 8.14 ± 0.17 mg GAE/g DW, respectively). The TF content of *E. fragilis* CEE and its fractions was evaluated by aluminum chloride colorimetric assay using quercetin as a standard (y = 0.0295x + 0.0361; R^2^ = 0.9986) (Table 4). Similarly to the TP content, the same results were observed in the TF content with the highest and lowest content being detected in the EAF (10.50 ± 0.11 mg QE/g of DW) and HF (1.65 ± 0.13 mg QE/g of DW), respectively. The TF content is arranged as the following sequence: EAF > WBF > DMF > CEE > WF > HF. Interestingly, the TP and TF contents in EAF were 2.18 and 3.59-fold higher than that of the CEE, suggesting that ethyl acetate may be the appropriate solvent to concentrate more available phenolics and flavonoids compounds during the CEE fractionation. Our data concerning TP and TF contents are consistent to those of Yao et al. [27] who report that the EAF obtained from the medicinal plant Pyrola asarifolia had the highest levels of phenolics and flavonoids compared to the other fractions (petroleum ether, n-butanol, and water). In their study, Bhardwaj et al. [28] also reported similar results when using several solvents with increasing polarity (n-hexane, chloroform, ethyl acetate, and n-butanol) in the splitting of a medicinal plant Codonopsis clematidea. According to these authors, the EAF has the highest TP and TF contents distantly followed by the n-butanol, chloroform, and hexane ones. This significant variation in the extraction yields, composition, and purity phenolic compounds between the fractions is probably due to the differences in the polarity of constituents found in plant materials, their chemical structure, their polymerization degree, and their interaction with each other [29].

### 2.6. In Vitro Antioxidant Activity

#### 2.6.1. DPPH^•^ Scavenging Activity 

DPPH^•^ is one among few stable free radicals that is widely used to investigate the antioxidant potential of plant extracts [30]. An extract’s scavenger potential is frequently associated with its ability to scavenge stable free radicals, which is due to its hydrogen-donating ability. 

**Figure 2:**
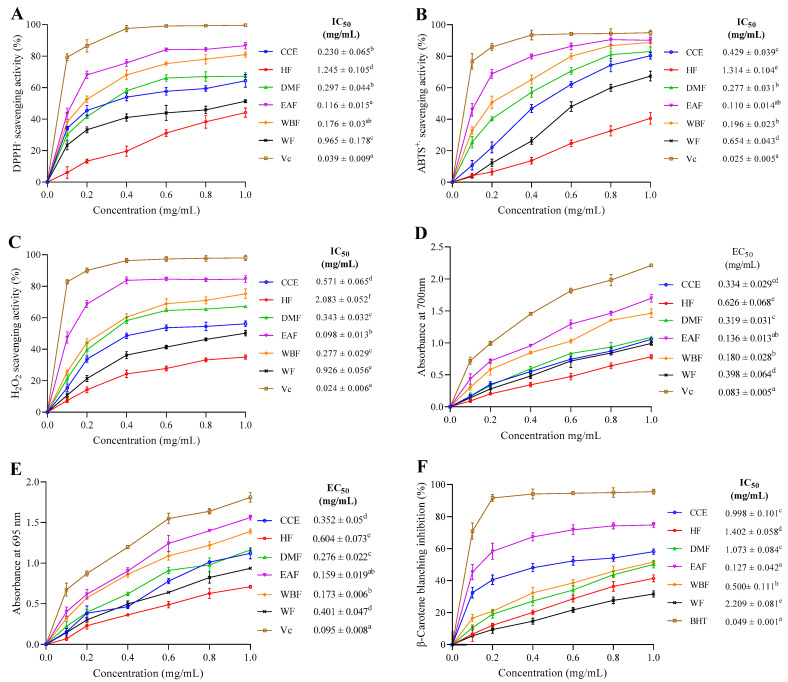
Antioxidant capacities of the crude ethanol extract obtained under optimum extraction conditions and its corresponding fractions from *E. fragilis*. (**A**) DPPH; (**B**) ABTS; (**C**) H_2_O_2_; (**D**) reducing power; (**E**) TAC; and (**F**) β-Carotene blanching inhibition activities. All the data were expressed in mean ± standard deviation (SD) for three independent experiments. Means with different superscript letter differ (*p* < 0.05), as tested by one-way ANOVA.

The scavenging capacity of CEE/fractions as well as VC at different doses on DPPH^•^ free radical was studied. Figure 2A shows that all fractions exhibited obvious DPPH^•^ scavenging activity in a dose-dependent fashion in the range of 0.1 to 1 mg/mL. At a dose of 1 mg/mL, 64.32, 44.10, 67.24, 86.63, 80.95, 51.37, and 99.62% of DPPH^•^ radical were quenched by CEE, HF, DMF, EAF, WBF, WF and VC, respectively. The lowest IC_50_ for scavenging DPPH^•^ among all fractions was displayed by EAF (0.116 ± 0.015 mg/mL) proceeded by WBF (0.175. ± 0.03 mg/mL), CEE (0.23 ± 0.065 mg/mL), and DMF (0.297 ± 0.044 mg/mL). WF and HF showed comparatively higher IC_50_ values (0.964 ± 0.178 and 1.245 ± 0.105 mg/mL), respectively. 

Observed differentials in the scavenging activities of the fractions against the DPPH^•^ radical may be assigned to the structural characteristics and the amount of phenolic compounds present in each fraction. Similar results were observed in the medicinal plant Liquidambar formosana Hance leaf, since the EAF was more effective than the other fractions (dichloromethane, n-butanol and of water fractions) [31]. As a standard molecule, ascorbic acid (VC) displayed the lowest IC_50_ value (0.039 ± 0.009 mg/mL) in comparison to all fractions. The scavenging potential is in decreasing order of VC > EAF > WBF > DMF > CEE > WF > HF. 

The IC_50_ value for DPPH^•^ radical scavenging was significantly positive correlated with both TP content (r = 0.963; *p* < 0.01) and TF content (r = 0.949; *p* < 0.01) as presented in Table 5. Therefore, the discovered antioxidant activity suggested that EAF can be a source of numerous natural compounds with antioxidant properties that can act as hydrogen donors to terminate the process of oxidation by converting the free radicals to their stable forms.

#### 2.6.2. ABTS^•+^ Scavenging Activity

The ABTS^•+^ scavenging assay is extensively used as an index to inform and investigate the antioxidant capacity of pure compounds as well as natural extracts [32,33]. The fading of the bleu/green color of the ABTS^•+^ chromophore at 734 in the presence of plant extract may indicate an antioxidant activity. 

As shown in Figure 2B, the scavenging curve of CEE/fractions on ABTS^•+^ exhibited an upward trend as the concentration increased. At 1 mg/mL, the scavenging rates of CEE, HF, DMF, WBF, EAF, WF, and VC were 80.31, 40.54, 88.76, 90.11, 82.97, 67.36, and 94.90%, respectively. Among all fractions, the minimum IC_50_ value was showed by EAF (0.110 ± 0.014 mg/mL) proceeded by DMF (0.196 ± 0.023 mg/mL), WBF (0.277 ± 0.031 mg/mL), CEE (0.429 ± 0.039 mg/mL), and WF (0.654 ± 0.043 mg/mL), whereas HF displayed the highest IC_50_ value (1.314 ± 0.104 mg/mL). Collectively, this finding was in agreement with that of Kaewseejan and Siriamornpun [29]. Comparison with the inhibitory capacity of VC (0.025 ± 0.005 mg/mL) showed that the scavenging activity of CEE and its fractions was slightly weak. 

As presented in Table 5, a highly significant correlation of ABTS^•+^ radical scavenging with TP content (r = 0.921; *p* < 0.01), and a significant correlation with TF content (r = 0.891; *p* < 0.05), were shown. This finding confirms the results obtained for the DPPH^•^ scavenging assay, proving the capacity of EAF to scavenge free radicals.

#### 2.6.3. H_2_O_2_ Scavenging Activity 

Being a chief contributor to oxidative stress, H_2_O_2_ diffuses readily through cells across the membrane as a messenger molecule [34]. Taken together, H_2_O_2_ itself is not dangerous [35], but it can react with Fe^2+^ through Fenton reaction and gives rise to the highly reactive hydroxyl radical (OH^•^) [36]. Thus, it is the most damaging of the ROS to biomolecules. It is therefore necessary to evaluate the ability of CCE and its various fractions to scavenging H_2_O_2_. 

Scavenging activities of CEE/fractions as well as standard antioxidant were presented in Figure 2C. Notably, all extracts showed a strong scavenging activity on H_2_O_2_ that increased with the increase of sample doses ranging from 0.1 to 1 mg/mL. Moreover, the H_2_O_2_ scavenging potential decreased in the order of EAF > WBF > DMF > CEE > WF > HF, and the corresponding scavenging abilities at 1.0 mg/mL were 84.56, 75.24, 67.17, 56.18, 50.19, and 34.95%, respectively, which were much lower than that of VC (98.03%). 

Among all fractions, EAF showed the lowest IC_50_ value (IC_50_ = 0.098 ± 0.013 mg/mL), and it was significantly higher than that of VC (0.024 ± 0.006 mg/mL). This may be due to the presence of high phenolic and flavonoid compounds in the EAF, which are widely known to play a crucial role as antioxidants in biological systems. In our recent study, we demonstrated the capacity of the EAF from *E. fragilis* to protect Tetrahymena pyriformis against H_2_O_2_-induced oxidative damage [37]. 

Our data analysis depicted that, as for the DPPH^•^ and ABTS^•+^ results, a strong positive correlation of the IC_50_ value for H_2_O_2_ scavenging was noted with both TP content (r = 0.926; *p* < 0.01) and TF content (r = 0.934; *p* < 0.01) (Table 5). In their study, Sroka and Cisowski [38] reported also a positive correlation between phenolic compounds with H_2_O_2_-scavenging ability. According to these authors, the H_2_O_2_-scavenging depended strongly on the number, positions, and the model of substitution of OH bonded to the aromatic ring of phenolic compounds.

#### 2.6.4. Reducing Power 

The reducing power of an extract acts as an indicator of its potential antioxidant activity [39,40]. The antioxidant potential is estimated by the capacity of antioxidants to reduce iron (Fe^3+^) in ferric chloride to ferrous (Fe^2+^). Generally, the reducing properties are attributed to the presence in plant extracts of reductones, which are recognized to exert their action by breaking the free radical chain by donating a hydrogen atom [41]. 

Figure 2D shows the dose response curve for the reducing power of *E. fragilis* extracts. It is known that the higher the absorbance at 700 nm, the greater the reduction ability. In the current study, CEE and its five fractions exhibited considerable reducing power in a concentration-dependent manner. 

The EAF showed maximum antioxidant activity (EC_50_ = 0.136 ± 0.013 mg/mL) in comparison to VC (EC_50_ = 0.083 ± 0.005 mg/mL). This was closely followed by WBF (EC50 = 0.180 ± 0.028 mg/mL), DMF (EC50 = 0.319 ± 0.031 mg/mL), CEE (EC50 = 0.334 ± 0.029 mg/mL), and WF (EC_50_ = 0.398 ± 0.064 mg/mL). HF trailed behind showing minimum reducing power activity (EC_50_ = 0.626 ± 0.068 mg/mL) compared to other fractions. These findings suggest that EAF may contain various individual compounds that have an effective and potent reducing power activity. Scientists have found that ethyl acetate extracts might serve as strong antioxidants [42]. 

Positive correlation was observed between reducing power and both TP and TF contents (r = 0.975; *p* < 0.01 and r = 0.987; *p* < 0.01; respectively). These correlations confirmed the contribution of phenolic compounds in the reducing power activity. 

#### 2.6.5. Phosphomolybdenum Assay 

TAC of the CEE and its fractions as well as VC were evaluated using the phosphomolybdenum assay and expressed as EC_50_, which is the concentration providing 0.5 of absorbance. The method was based on the reduction of Mo (VI) to Mo (V) by extracts to form of a green phosphate/Mo (V) complex at acidic pH with a maximum absorption at 695 nm [43]. 

As shown in Figure 2E, the total antioxidant capacity of CCE/fractions and VC correlated well with increasing concentrations in the range of 0.1 to 1 mg/mL. Of the CEE fractions, the EC_50_ values ranged from 0.159 ± 0.019 to 0.604 ± 0.073 mg/mL, with a descending order of EAF > WBF > DMF > CEE >WF > HF (*p* < 0.05), which indicates that EAF and HF had the highest and lowest antioxidant activity, respectively. This activity could be due to the presence in EAF of various phenolic compounds that might possess an antioxidant activity. VC, which is the positive control, displayed the lowest EC_50_ value (0.095 ± 0.008 mg/mL) in comparison to all fractions. 

A highly significant correlation was observed between total antioxidant activity and both TP content and TF contents (r = 0.978 for both; *p* < 0.01) and is shown as presented in Table 5. 

#### 2.6.6. β-Carotene–Linoleate Model System

In the β-carotene-linoleic acid model, the highly unsaturated β-carotene molecules undergo rapid discoloration due to linoleate free radicals generated by the oxidation of linoleic acid [44]. Supplementation with antioxidant could minimize β-carotene oxidation by neutralizing linoleate free radicals, and thus inhibiting β-carotene bleaching [45]. 

Antioxidant activity of CCE/fractions and BHT, as measured by β-carotene–linoleate model, are shown in Figure 2F. All tested extracts showed concentration-dependent scavenging activity. The EAF, which contained the highest amount of phenolics and flavonoids contents, showed a significant effect in inhibiting β-carotene bleaching, reaching 74.75% at a concentration of 1 mg/mL. WBF, CCE, DMF, HF, and WF inhibited the oxidation of β-carotene by 51.61, 58.12, 50.09, 41.39, and 31.66% respectively, at the same concentration. Overall, decreasing antioxidant activity was depicted as EAF > WBF > CEE > DMF > HF > WF. 

The EAF displayed the minimum IC_50_ value (IC_50_ = 0.127 ± 0.042 mg/mL) in comparison to BHT (IC_50_ = 0.049 ± 0.001 mg/mL). WBF, CCE, DMF, WF, and HF displayed comparatively higher IC_50_ values of 0.5 ± 0.111 mg/mL, 0.998 ± 0.101 mg/mL, 1.073 ± 0.084 mg/mL, 1.402 ± 0.058 mg/mL, and 2.209 ± 0.081 mg/mL, respectively. 

A significant correlation in a positive manner was observed between β-carotene oxidation scavenging and both TP content (r = 0.850; *p* < 0.05) and TF content (r = 0.885; *p* < 0.05) (Table 5). This result suggests that EAF may contain some antioxidants that can inhibit the formation of hydroperoxide and stop the radical-chain reaction [46].

The involvement of reactive oxygen species (ROS) in several pathological situation has been growing recently. Bioactive compounds are gaining interest thanks to their potent antioxidant activity, but their complexity imposes the development of many methods to evaluate the antioxidant activity and the effectiveness of these chemical compounds. Thus, in this study, CEE and its fractions have been investigated for their antioxidant potential using six assays: DPPH, ABTS, H_2_O_2_, RP, TAC, and β-carotene. Hence, we showed that the potent antioxidant activities of CEE and its fractions exhibited higher scavenging activities, and this may strongly be due to their composition from phenolic acid and flavonoids such as gallic acid, rutin, and quercetin.

### 2.7. Antiglycation Activity 

#### 2.7.1. UV-Visible Analysis 

**Figure 3:**
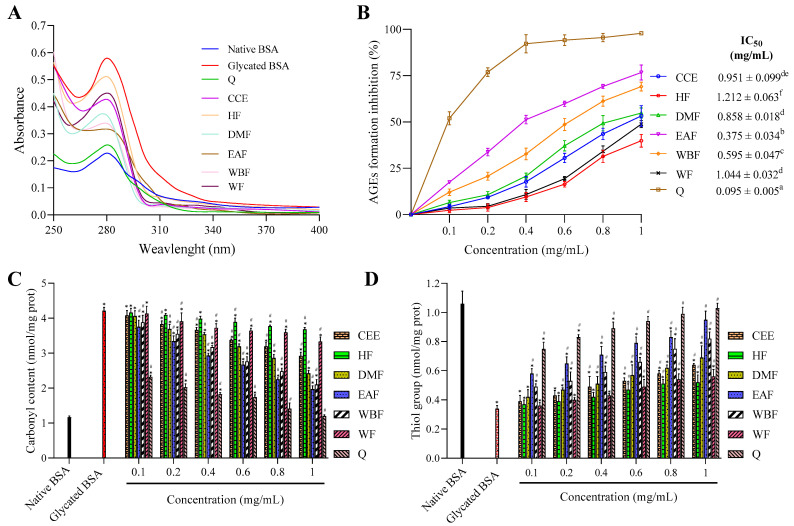
The effect of CEE and its fractions from *E. fragilis* extract on the (**A**) UV–vis absorption spectrum, (**B**) AGEs formation, (**C**) carbonyl content, and (**D**) the level of thiol group in glucose-glycated BSA. All values are expressed as means ± SD, *n* = 3. Means without a common superscript letter differ (*p*  <  0.05), as analyzed by one-way ANOVA. In the same graph, bars with * represent significantly different from native BSA at *p* < 0.05 and bars with # represent significantly different from glycated BSA at *p* < 0.05.

The UV–vis spectrum is a fast, consistent, and simple technique commonly used to detect protein conformational changes and complex formation. The absorption spectra of native and glycated BSA incubated for 15 days in the presence or absence of CEE/fractions as well as quercetin (positive control) are presented in Figure 3A. 

It was clearly showed that the native BSA exhibits a characteristic peak at $\lambda_{280}$ nm, which is mostly due to the aromatic amino acids, including tyrosine, tryptophan, and phenylalanine [47]. 

Upon modification with glucose, absorbance at $\lambda_{280}$ nm was 60.57% more hyperchromic than native BSA. The increased absorption intensity at $\lambda_{280}$ nm can be attributed to glycation-induced unfolding of protein helix, which can affect its normal physiological function. 

Treatment with CEE/fractions reduced significantly the absorbance at $\lambda_{280}$ nm compared to glycated BSA, and this reduction varied markedly between fractions according to the solvent polarity. Overall, descending antiglycation activity was portrayed as EAF > WBF > DMF > CCE > WF > HF, which were 1.82, 1.71, 1.57, 1.35, 1.28, and 1.13-fold lower than glycated BSA. Nevertheless, this activity was markedly lower than that of quercetin used as positive control (2.24-fold lower than glycated BSA). So, it can be clearly concluded from absorption studies that EAF from *E. fragilis* possess protective effect against BSA unfolding induced by protein glycation. 

#### 2.7.2. Inhibition of Protein Glycation in the BSA-Glu Model

AGEs are heterogeneous group of compounds with fluorescence characteristic at $\lambda_{440}$ nm when excited at $\lambda_{370}$ nm. The ability of CEE and its various fractions from *E. fragilis* to inhibit AGEs formation was evaluated using the BSA–glucose assay, and the results are presented in Figure 3B. 

As evidence from Figure 3B, AGEs inhibition rate of all tested samples exhibited an upward trend with the increase of concentrations. At 1 mg/mL, CCE, HF, DMF, EAF, WBF, WF, and quercetin inhibited AGEs formation by 53.26, 39.83, 54.82, 76.68, 69.09, 48.83, and 97.84%, respectively, after incubation for 15 days. The EAF (IC_50_ = 0.375 ± 0.034 mg/mL) was the most effective AGEs inhibitor among all fractions, followed by WBF, DMF, CCE, and WF with IC_50_ values of 0.595 ± 0.047, 0.857 ± 0.018, 0.951 ± 0.099, and 1.044 ± 0.032 mg/mL, respectively. HF showed the weakest activity with the IC_50_ value as 1.212 ± 0.063 mg/mL. 

The high antiglycation potential of EAF could be due to the high amount of phenolic and flavonoid contents, which have been described as very good inhibitors of AGEs formation [48]. Higher antiglycation of the EAF of Liquidambar formosana Hance leaf extract was also reported by Zhang et al. [31] as compared with that of its dichloromethane, n-butanol, and water fractions. 

As given in Table 5, AGEs inhibition was strongly corelated in a positive manner to both TF (r = 0.950; *p* < 0.01) and TP (r = 0.972; *p* < 0.01) contents, which were in line with previously reported studies [2,29]. The AGEs inhibition was also correlated in a positive way to DPPH^•^, ABTS^•+^, H_2_O_2_, reducing power, TAC, and β-carotene assays with r = 0.930, r = 0.914, r = 0.983, r = 0.975, r = 0.923, and r = 0.963 (*p* < 0.01), respectively. These reflected that AGEs inhibition is linked to the efficiency of primary antioxidants [6]. Kaewseejan and Siriamornpun [29] also reported that phenolic compounds prevented the formation of AGEs through its free radicals scavenging and antioxidant capacities. 

#### 2.7.3. Effects on Glycation-Induced Protein Oxidation 

Glycation of proteins (Maillard reaction) is a reaction started by the covalent attachment of a reducing sugar to an amino group of proteins (mainly lysine and arginine residues), which leads to producing an unstable and reversible product i.e., Schiff’s base that further undergoes Amadori rearrangement to form more stable ketoamines named Amadori products. Subsequently, Amadori products undergo enediol reaction to produce carbonylated proteins [48]. The degradation of these ketoamines could generate free radicals such as superoxide radicals, which further converted into HO^•^ via Fenton reaction, causing oxidative and cellular damage [49]. 

Protein oxidation is accompanied by carbonyl protein formation and loss of protein thiols, which are often employed as protein oxidation indicators [50]. As given in Figure 3C, the level of carbonyl content in native BSA was 1.16 ± 0.04 nmol/mg protein, which was increased to more than 3.62-fold (4.21 ± 0.10 nmol/mg protein) upon glycation. The treatment with CEE and its various fractions reduced the level of carbonyl content with the increase of samples concentrations ranging from 0.1 to 1 mg/mL. Furthermore, the inhibition effect of quercetin on the formation of carbonyl proteins was stronger than that of all fractions at every concentration point. When the concentration was 1 mg/mL, CEE, HF, DMF, EAF, WBF, WF, and quercetin decreased the level of carbonyl content by 42.29, 17.37, 58.68, 73.44, 69.17, 28.84, and 98.68%, respectively, compared to native BSA. Overall descending, the inhibition of carbonyl content formation was portrayed as Q > EAF > WBF > DMF > CCE > WF > HF. 

The effects of CEE/fractions on glycation-induced protein thiol oxidation are presented in Figure 3D. In native BSA, the level of protein thiol was 1.06 ± 0.086 nmol/mg protein, which was decreased by more than three-fold (0.34 ± 0.021 nmol/mg protein) in glycated protein. In the presence of CEE/fractions, the level of the thiol group was significantly increased in a dose-dependent manner ranging from 0.1 to 1 mg/mL. Moreover, the level of protein thiol increased in the following order: HF < WF < CEE < DMF < WBF < EAF, and its corresponding levels at 1 mg/mL were 0.52 ± 0.05, 0.56 ± 0.048, 0.64 ± 0.01, 0.69 ± 0.045, 0.82 ± 0.081, and 0.95 ± 0.059 nmol/mg protein, respectively, which were less effective than quercetin (1.03 ± 0.033 nmol/mg protein). 

Similar results were observed in the plant *Teucrium polium*, since the EAF was more effective than the other fractions (diethyl ether and water fractions) against glycation-mediated protein oxidation [51]. In their study, Golshahi and Bahramikia [52] also reported similar results when using several solvents with increasing polarity (diethyl ether and water) in the splitting of a medicinal plant *Trachyspermum copticum*. According to these authors, the EAF has the most potent protective effect against glycation-mediated protein oxidation, distantly followed by the diethyl ether and water ones. This demonstrates the presence in EAF of such compounds that might possess a preventive effect against hyperglycemia-induced oxidative damages to protein, which is believed to occur under the glycoxidation processes by reducing protein carbonyl formation and protecting protein thiols from oxidation as suggested by data.

### 2.8. Identified Phenolic Compounds in the EAF 

**Figure 4:**
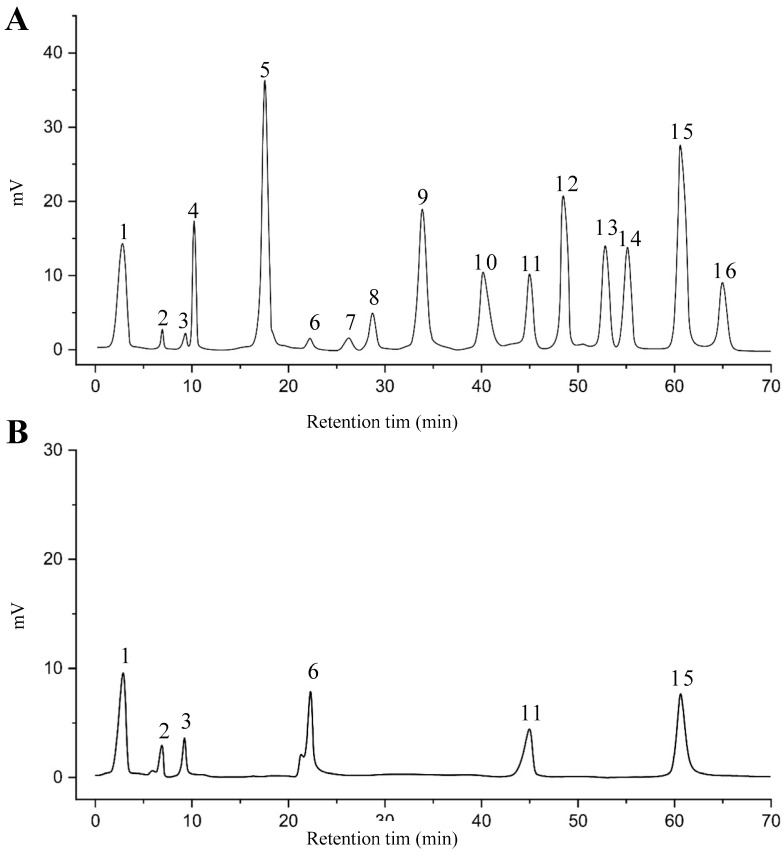
HPLC chromatogram of (**A**) 16 available polyphenol standards and (**B**) EAF from *E. fragilis*. Peak numbers correspond to chemical compounds gallic acid (retention time (R_t_) = 2.76 min, peak 1), vanillic acid (R_t_ = 6.94 min, peak 2), caffeic acid (R_t_ = 9.34 min, peak 3), syringic acid (R_t_ = 10.23 min, peak 4), catechin (R_t_ = 17.52 min, peak 5), ferulic acid (R_t_ = 22.28 min, peak 6), *p*-coumaric acid (R_t_ = 26.23 min, peak 7), sinapic acid (R_t_ = 28.67 min, peak 8), chlorogenic acid (R_t_ = 33.81 min, peak 9), isoquercitrin (R_t_ = 40.06 min, peak 10), rutin (R_t_ = 44.95 min, peak 11), quercetol (R_t_ = 48.43 min, peak 12), luteolin (R_t_ = 52.76 min, peak 13), kaempferol (R_t_ = 55.11 min, peak 14), quercetin (R_t_ = 60.51 min, peak 15), and apigenin (R_t_ = 64.94 min, peak 16).

The EAF, which showed the highest biological activity from other fractions, was selected for the identification of its main bioactive compounds by RP-HPLC. A total number of six compounds were identified by comparing their retention time with those of reference standards. The identified phenolic compounds are presented in Figure 4. Gallic, vanillic, caffeic, and ferulic acids were identified as phenolic acids, whereas only two compounds, namely rutin and quercetin, were identified as flavonoids. According to a study by Soumaya et al. [53], ferulic acid, luteolin-7-*O*-glucoside, myricetin, and kaempferol 3-*O*-rutinoside were identified as present in the EAF of aerial parts of Tunisian *E. fragilis*, whereas the presence of rutin, quercetin, gallic acid, and caffic acid was only detected for the first time in our study. The disparity in the chemical composition of the EAF obtained from the same plant species can differ in different parts of a plant, the stage of plant development, the growth conditions (e.g., soil, light, temperature, water, humidity, and fertilizers), harvesting time, the drying system, and the extraction procedure [54]. The obtained results from the phytochemical fingerprint profile showed a good wealth of *E. fragilis* that had several phenolic compounds that are considered major contributors to the free radicals scavenging and antioxidant activities [55]. In addition, these compounds are known for their powerful antiglycation capacities [48]. Several studies have revealed the direct connection between antioxidant activities of phenolic compounds and their antiglycation capacities. 

### 2.9. Molecular Docking Study of Identified Compounds 

**Figure 5:**
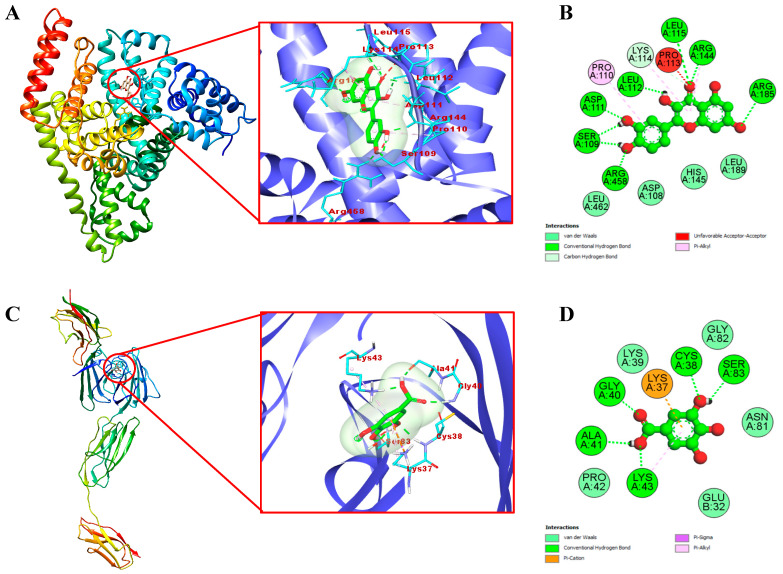
The 3D view of the binding mode between quercetin (**A**) and gallic acid (**C**) with BSA and RAGE, respectively. The 2D detailed view showed the interaction between quercetin (**B**) and gallic acid (**D**) with neighboring residues of BSA and RAGE, respectively.

To clearly visualize the detailed mechanism by which the identified compounds in the EAF of *E. fragilis* bind with BSA and RAGE, we performed a molecular docking study. Docking results are presented in Table 6, while interactions between the most active compound and targets are shown in Figure 5. Results showed that quercetin snugly fitted into the binding site, located in the hydrophobic cavity of subdomain IB of BSA with the lowest binding energy of −7.7 kcal/mol (Figure 5A). In contrast, lesser binding energy was obtained with ferulic acid, vanillic acid, caffeic acid, gallic acid, and rutin (−6.35, −6.05, −5.84, −5.25 and −4.41 kcal/mol, respectively). Usually, a high degree of negativity of binding energy is more effective and the compound would be used for controlling the glycation processes. From Figure 5B, it is clear that quercetin form eight hydrogen bonds with SER109, ASP111, LEU112, LEU115, ARG144, ARG185, and ARG458 of BSA, and four hydrophobic interactions mediated by the aliphatic amino acids (PRO110, PRO113, LYS114, and ARG144). Also, four amino acids (ASP108, HIS145, LEU189, and LEU462) surrounding quercetin interacted via van der Waal’s forces. It has been reported that lysine and arginine are the main amino acid residues involved in the glycation process [56]. Therefore, masking of quercetin to lysine and arginine residues could be one of the possible mechanisms of *E. fragilis* to inhibit protein glycation at an initial stage. 

**Figure 6:**
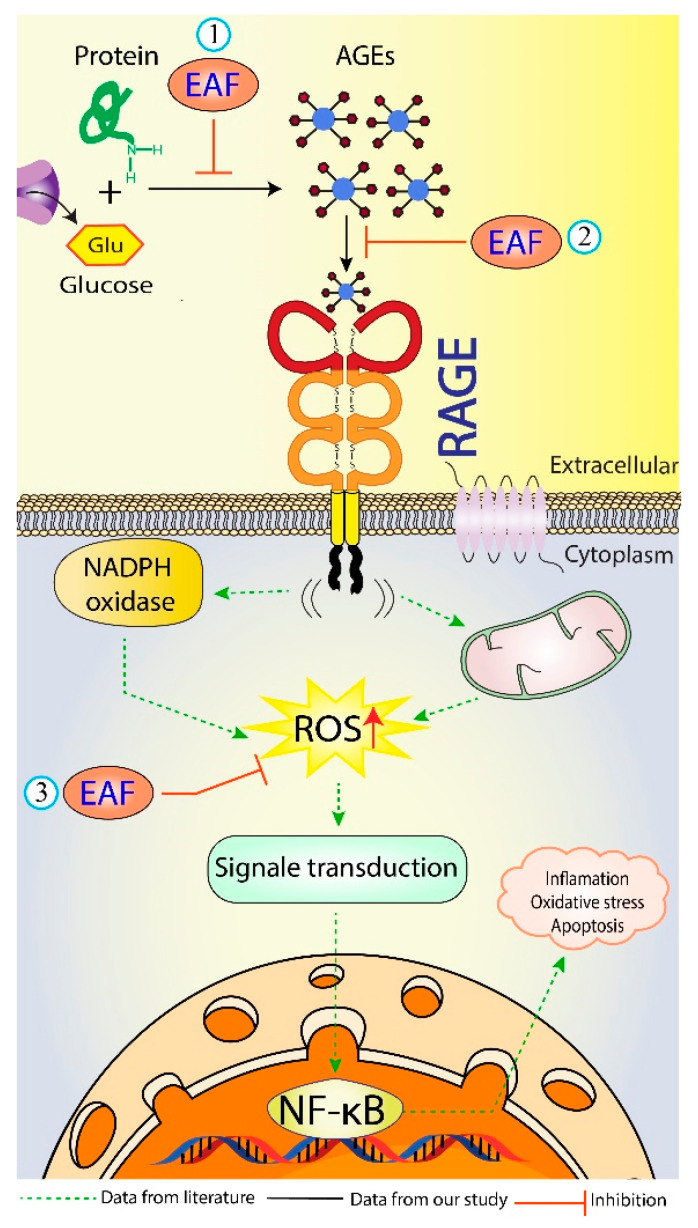
Schematic representation showing the possible antiglycation mechanisms of EAF of *E. fragilis*. (1) Inhibition of harmful AGEs formation, (2) Blocking of AGEs-RAGE interaction, and (3) Inhibition of ROS formation during glycation. AGEs = advanced glycation end products; RAGE = receptor of AGEs; ROS = reactive oxygen species.

Engagement of AGEs products with RAGE are known to trigger, through ROS formation via NADPH oxidase and mitochondria [57], the activation of multiple intracellular signaling pathways (including JAK/STAT, phosphoinositol-3 kinase, rho GTPases, SAPK/JNK MAP kinases, p38 and erk1/2 (p44/p42) MAP kinases), and culminating in the activation of the NF-κB transcription factors [58], leading to the pathogenesis of diabetes and aging-associated disorders [5]. Therefore, blocking the AGEs–RAGE interactions can repress stress-provoking signals transduction, which is considered a therapeutic strategy of inhibiting glycation at a later stage. Docking results with RAGE, as shown in Table 6, proved that gallic acid has the highest docking score (ΔG= −6.8 kcal/mol) in comparison to those of vanillic acid, ferulic acid, caffeic acid, quercetin, and rutin (−6.68, −5.94, −5.89, −5.58 and −4.89 kcal/mol, respectively). Moreover, 2D modeling of gallic acid and RAGE showed that gallic acid formed five conventional hydrogen bonds with CYS38, GLY40, ALA41, LYS43, and SER83 of RAGE (Figure 5C,D). Also, LYS37 and LYS43 were responsible for the hydrophobic interactions of gallic acid with RAGE. Five amino acids surrounding gallic acid (GLU32, LYS39, PRO42, ASN81, and GLY82) were attached by van der Waal’s forces, thereby stabilizing the gallic acid-RAGE complex by providing a strong cohesive environment. In summary, the current study has shown the efficient interaction of certain bioactive compounds in the EAF of *E. fragilis* with the target proteins of BSA and RAGE. *E. fragilis* could be a source of potential competitors to glucose and AGEs, which might resist their binding towards BSA and RAGE, respectively, and therefore reducing the subsequent development of oxidative stress and inflammation (Figure 6).

## 3. Materials and Methods

### 3.1. Chemicals and Reagents 

2,2-azinobis-(3-ethylbenzothiazoline-6-sulphonic acid) (ABTS), butylated hydroxytoluene (BHT), sodium azide, guanidine hydrochloride, tween-40, 1,1-diphenyl-2-picrylhydrazyl (DPPH), Folin Ciocalteau reagent, β-carotene, linoleic acid, glucose, 2,4- dinitrophenylhydrazine (DNPH), 5,5’-Dithiobis-(2-Nitrobenzoic Acid) (DTNB), ammonium molybdate, and polyphenols standards were purchased from Sigma-Aldrich (St. Louis, MO, USA). Hydrogen peroxide (H_2_O_2_) was purchased from Fluka (Basel, Switzerland). Aluminum chloride (AlCl₃), sodium carbonate (Na₂CO₃), ferric chloride (FeCl_3_), potassium persulphate (K_2_S_2_O_8_), potassium ferricyanide (K_3_Fe(CN)_6_), sulfuric acid, and all solvents were obtained from Merck Life Science (Darmstadt, Germany). Ascorbic acid and trichloracetic acid (TCA) were obtained from Scharlau (Barcelona, Spain). 

### 3.2. Plant Materials 

*E. fragilis* (aerial parts) was collected from Dour Lagfifat, Oulad Teima, Taroudant, Morocco (latitude, 30°24′0″ N; longitude, 9°12′36″ W) during May 2019. It was identified by Professor Najat ELKHIATI, a botanist from our institute, where a collection of voucher specimens was deposited. The plant was rinsed with distillated water, air dried, powdered in a blender, and stored at 4°C until use. 

### 3.3. Experimental Design 

#### 3.3.1. Selection of Variables 

Many parameters are known to have significant effects on phenolic compounds extraction, such as the type solvent, solvent concentration, extraction time, and extraction temperature [59]. Different solvents such as acetone, methanol, and ethanol are suitable for the extraction of different phenolic compounds [16], but ethanol was selected as the solvent in this study, due to its edible safety and green manufacturing [60]. Therefore, all factors, including the ethanol concentration ($X_{1}$), extraction temperature ($X_{2}$), and extraction time ($X_{3}$) were selected as variables.

#### 3.3.2. BBD for Extraction Optimization 

An optimization procedure was developed using RSM to determine the effects of extraction factors and choose the optimum experimental extraction conditions of *E. fragilis* phenolic compound. A three-level, three-factor BBD was undertaken to investigate the impact of three independent factors including $X_{1}$ (ethanol concentration, %), $X_{2}$ (extraction temperature, °C), and $X_{3}$ (extraction time, h) on TP and TF contents of *E. fragilis* extracts [17]. For optimization purposes, a total number of 15 trials including three center points were carried out randomly (Table 1). Based on our preliminary single factor experiment (data not shown), all variables were set at three levels (−1, 0 and +1), with $X_{1}$ (40, 60 and 80%), $X_{2}$ (25, 42.5 and 60 °C), and $X_{3}$ (6, 15 and 24 h) (Table 1). The following second order polynomial equation (Equation (1)) was used to fit the response variables:

(3) $$Y=\beta_{0}\;+\;\overset{3}{\underset{i=1}{\sum }}\beta_{i}X_{i}\;+\;\overset{3}{\underset{i=1}{\sum }}\beta_{\mathrm{ii}}X_{i}^{2}\;+\;\overset{2}{\underset{i=1}{\sum }}\overset{3}{\underset{j=i+1}{\sum }}\beta_{\mathrm{ij}}X_{i}X_{j}\;$$

where Y is the predicted response; $\beta_{0}$, $\beta_{i}$, $\beta_{\mathrm{ii}},$ and $\beta_{\mathrm{ij}}$ are the regression coefficients for intercept, linear, quadratic and interaction terms, respectively; and $X_{i}$, and $X_{j}$ are the independent variables (*i* ≠ *j*).

#### 3.3.3. Extraction Procedure 

The powdered sample (10 g) was extracted by maceration method in a designed ethanol concentration (40–80%; 1:10, *w*/*v*), at varying temperatures (25–60 °C) for various periods (6–24 h) on an orbital shaker incubator (160 rpm). Gauze and Whatman filter paper no. 1 were used to remove the insoluble mass. The filtrate was then dried at 40 °C under low pressure using R-3 Rotavapor (Büchi) to yield the crude ethanolic extract (CEE).

### 3.4. Fractionation of the CEE Obtained under Optimum Condition 

The CEE obtained under optimum condition was solubilized in distilled water (100 mL), and a liquid–liquid extraction was performed with various solvents of increasing polarity to yield hexane fraction (HF, 3 × 100 mL), dichloromethane fraction (DMF, 3 × 100 mL), ethyl acetate fraction (EAF, 3 × 100 mL), water-saturated n-butanol fraction (WBF, 3 × 100 mL), and the remaining water fraction (WF). These fractions were then filtered and dried as described above, and the extraction yield was recorded according to Equation (2):

(4) $$\mathrm{Extraction}\;\mathrm{yield}\;\left(\%\right)=\left(\frac{W_{0}}{W_{1}}\right)\times 100$$

Were $W_{0}$ and $W_{1}$ are the weight of dried CEE/fractions and initial weight of *E. fragilis* powder; respectively.

### 3.5. Phytochemical Analysis 

The spectrophotometric techniques used to evaluate the phytochemical contents of *E. fragilis* extracts are detailed in the Supplementary Materials [61,62]. 

### 3.6. Biological Activities 

Details of the antioxidant [43,63,64,65,66,67] and antiglycation activities [68,69,70] tests in vitro were given in the Supplementary Materials. 

### 3.7. RP-HPLC Analysis of EAF 

Analysis of phenolic compounds in the EAF was performed with Agilent 1100 (Agilent technologies, Santa Clara, CA, USA) equipped with a ZORBAX Eclipse SB-C18 reversed-phase analytical column of 100 × 4 mm and 3.5 µm particle size [71]. Column temperature was kept constant at 48 °C. An isocratic elution with acetonitrile, 0.1% acetic acid in water (12:88, *v*/*v*) as mobile phase, and 1 mL/min flow rate ensured good separation of polyphenols in the EAF of *E. fragilis*. The injected volume was 10 μL and chromatograms were measured at 330 nm. The retention times of phenolic compounds in the EAF were compared to those of pure available standards to identify them. 

### 3.8. Molecular Docking 

AutoDock tools (ADT) version 1.5.6 was used to perform molecular docking study. SDF format of all compounds was obtained from PubChem database and then converted to 3D pdb file using Open Babel GUI (version 2.4.1). The crystal structures of BSA (PDB ID: 4OR0) and RAGE (PDB ID: 4LP5) were collected from the RCSB Protein Data Bank (PDB). Briefly, the proteins were firstly prepared for docking by: (i) removing all heteroatoms and water molecules, (ii) adding polar hydrogen atoms, and (iii) assigning Kollman charges. The grid box dimension was set to x = 126, y = 126, z = 126 and x = 80, y = 80, z = 90 with grid center of x = 8.415, y = 21.626, z = 106.57 and x = 37.98, y = −43.581, z = 9.371 with a grid spacing of 0.375 Å created around the binding site of BSA and RAGE, respectively. The docking software was run 100 times using the Lamarckian Genetic Algorithm (LGA) to find the best binding pose. The ligand with the lowest binding energy score was chosen for further investigation. Discovery Studio software version 2020 (BIOVIA, San Diego, CA, USA) was used for visualizing docking results. 

### 3.9. Statistical Analysis 

Design-Expert software version 11^®^ (Stat-Ease Inc., and Minneapolis, MN, USA) was used to perform RSM. Data analysis was performed by one-way analysis of variance (ANOVA) followed by Duncan’s post-hoc test using SPSS 26.0 (IBM Co., USA); *p* < 0.05 was considered statistically significant. Pearson correlation analysis was conducted to investigate correlations between variables and their significance. All graphics were constructed using GraphPad Prism 7.0 software (San Diego, CA, USA). All experiments were conducted in triplicate and presented as mean values ± standard deviation (SD).

## 4. Conclusions

RSM with a BBD was employed to set the optimized parameters for extraction of the bioactive compounds from the Moroccan medicinal herb *E. fragilis*. The optimum ethanol concentration, extraction temperature, and extraction time were predicted for maximum extraction yield of phenolic compounds and showed to be 61.93%, 44.43 °C, and 15.84 h, respectively. The CEE obtained under optimum extraction conditions and its various fractions were analyzed for their TP and TF contents, as well as their antioxidant and antiglycation activities. The EAF fraction shows the highest TP and TF contents and the strongest antioxidant activities compared to other fractions. Also, the evaluation of several biomarkers such as UV-vis absorption spectrum, specific AGEs fluorescence, carbonyl content, and free thiols group, showed the greatest protective effect of EAF against glycation mediated by glucose. Furthermore, a significant positive relationship was observed between antioxidant capacities of phenolic compounds and their antiglycation activities. This indicates that phenolics compounds may be the main predominant components responsible for both antioxidant and antiglycation activities. The bioactive compounds in the EAF were characterized by RP-HPLC analysis and a total number of six compounds were identified. In silico molecular docking analysis also displayed an effective interaction between quercetin and gallic acid with BSA and RAGE as target proteins, respectively. Collectively, this study suggests that *E. fragilis* might be a potential source of natural bioactive compounds with powerful antioxidant and antiglycation activities and should be applied in the treatment and prevention of aging and glycation-associated complications. Further studies on bioactive compounds isolation and pharmacological screening (i.e., cytotoxicity study) need to be conducted to explore the phytochemistry and mechanisms of action of pharmacological properties of *E. fragilis*.

## Figures and Tables

**Table 1:** Levels and code of variable used for Box–Behnken design (BBD), and the observed responses at different experimental conditions.

Variable	Units	Symbol	Variable Levels		
			Low (−1)	Middle (0)	Hight (+1)
Ethanol concentration	%	X_1_	40	60	80
Extraction temperature	°C	X_2_	25	42.5	60
Extraction time	h	X_3_	6	15	24
Run	Extraction conditions			Experimental results	
	X_1_ (ethanol concentration, %)	X_2_ (extraction temperature, °C)	X_3_ (extraction time, h)	TP (mg GAE/g dw) *	TF (mg QE/g dw) *
1	40 (−1)	42.5 (0)	24 (+1)	12.41	2.82
2	40 (−1)	25 (−1)	15 (0)	11.59	2.75
3	60 (0)	42.5 (0)	15 (0)	15.26	2.98
4	60 (0)	42.5 (0)	15 (0)	15.39	2.96
5	60 (0)	60 (+1)	6 (−1)	11.57	2.73
6	80 (+1)	60 (+1)	15 (0)	13.21	2.78
7	60 (0)	60 (+1)	24 (+1)	13.65	2.81
8	40 (−1)	60 (+1)	15 (0)	13.67	2.84
9	60 (0)	25 (−1)	6 (−1)	12.18	2.72
10	80 (+1)	25 (−1)	15 (0)	13.39	2.86
11	60 (0)	42.5 (0)	15 (0)	15.26	2.98
12	80 (+1)	42.5 (0)	6 (−1)	12.41	2.81
13	60 (0)	25 (−1)	24 (+1)	10.31	2.77
14	80 (+1)	42.5 (0)	24 (+1)	13.19	2.79
15	40 (−1)	42.5 (0)	6 (−1)	12.13	2.69

* Experiments were performed in triplicate and the data were reported as means of three values.

**Table 2:** ANOVA results for total phenolics (TP) content and total flavonoids (TF) content.

Source	Total Phenolics (TP) Content (mg GAE/g _dw_)					Total Flavonoids (TF) Content (mg QE/g _dw_)				
	Sum of Squares	DF ^a^	Mean Square	*F*-Value	*p*-Value	Sum of Squares	DF	Mean Square	*F*-Value	*p*-Value
Model	30.2	9	3.36	84.96	<0.0001 ***	0.1172	9	0.0130	90.83	<0.0001 ***
X_1_ -Ethanol	0.72	1	0.7200	18.23	0.0079 **	0.0024	1	0.0024	17.09	0.0090 **
X_2_ -Temperature	2.68	1	2.68	67.84	0.0004 ***	0.0004	1	0.0004	3.14	0.1366 ^ns^
X_3_- Time	0.2016	1	0.2016	5.10	0.0734 ^ns^	0.0072	1	0.0072	50.23	0.0009 ***
X_1_^2^	2.77	1	2.77	70.01	0.0004 ***	0.0196	1	0.0196	136.96	<0.0001 ***
X_2_^2^	8.01	1	8.01	202.80	<0.0001 ***	0.0319	1	0.0319	222.40	<0.0001 ***
X_3_^2^	13.37	1	13.37	338.50	<0.0001 ***	0.0558	1	0.0558	389.20	<0.0001 ***
X_1_ X_2_	1.28	1	1.28	32.33	0.0023 **	0.0072	1	0.0072	50.41	0.0009 ***
X_1_ X_3_	0.0625	1	0.0625	1.58	0.2640 ^ns^	0.0056	1	0.0056	39.24	0.0015 **
X_2_ X_3_	3.90	1	3.90	98.75	0.0002 ***	0.0002	1	0.0002	1.57	0.2656 ^ns^
Residual	0.1975	5	0.0395			0.0007	5	0.0001		
Lack of fit	0.1862	3	0.0621	11.02	0.0843 ^ns^	0.0005	3	0.0002	1.13	0.5024 ^ns^
Pure error	0.0113	2	0.0056			0.0003	2	0.0001		
Cor Total	30.40	14				0.1179	14			
R^2^	0.9935					0.9939				
Adjusted R^2^	0.9818					0.9829				
Predicted R²	0.9012					0.9338				
C.V % ^b^	1.52					0.4246				
Adeq Precision	29.4772					28.7290				

^a^ Degree of freedom. ^b^ Coefficient of variation. Different superscripts (^ns^, **, ***) in columns indicate not significant at *p* > 0.05, significant at *p* < 0.05, *p* < 0.01 and *p* < 0.001; respectively.

**Table 3:** Experimental data of the validation of predicted values at optimal extraction conditions.

Extraction Variables			TP ^a^ (mg GAE/g of _dw_)		TF ^a^ (mg QE/g of _dw_)	
X_1_ (Ethanol Concentration, %)	X_2_ (Temperature, °C)	X_3_ (Time, h)	Predicted Value	Experimental Value ^b^	Predicted Value	Experimental Value ^b^
61.93	44.43	15.84	15.373	14.98 ± 0.29	2.975	2.92 ± 0.09

^a^ TP and TF represent total phenolic content and total flavonoid content, respectively. ^b^ Means of triplicate determination.

**Table 4:** Extraction yield, TP, and TF contents of CEE and its solvent fractions isolated from *E. fragilis*.

Fractions	Yield (%, *w*/*w*)	TP (mg GAE/g of _dw_)	TF (mg QE/ g of _dw_)
CEE	10.6 ± 0.98 ^d^	14.98 ± 0.29 ^c^	2.92 ± 0.09 ^b^
HF	0.78 ± 0.05 ^a^	8.04 ± 0.17 ^a^	1.65 ± 0.13 ^a^
DMF	1.34 ± 0.08 ^a,b^	19.21 ± 0.22 ^d^	4.29 ± 0.18 ^c^
EAF	0.93 ± 0.03 ^a^	32.78 ± 0.49 ^f^	10.50 ± 0.11 ^e^
WBF	2.04 ± 0.11 ^a,b,c^	25.02 ± 1.01 ^e^	7.64 ± 0.21 ^d^
WF	2.73 ± 0.17 ^c^	10.47 ± 0.71 ^b^	1.86 ± 0.28 ^a^

Values are expressed as mean ± SD of triplicate measurements. Means with different letters in the same column represent significant differences at *p* < 0.05.

**Table 5:** Pearson’s correlations between values obtained from each assay.

	TP	TF	DPPH	ABTS	H_2_O_2_	RP	TAC	β-carotene	AGEs
TP	1	0.986 **	0.963 **	0.921 **	0.926 **	0.975 **	0.978 **	0.850 *	0.950 **
TF		1	0.949 **	0.891 *	0.934 **	0.987 **	0.978 **	0.885 *	0.972 **
DPPH			1	0.873 *	0.908 **	0.952 **	0.924 **	0.860 *	0. 930 **
ABTS				1	0.960 **	0.866 *	0.835 *	0.892 *	0.914 *
H_2_O_2_					1	0.929 **	0.864 *	0.979 **	0.983 **
RP						1	0.979 **	0.884 *	0.975 **
TAC							1	0.787 ^ns^	0.923 **
β-carotene								1	0.963 **
Anti-AGEs									1

Different superscripts (^ns^, *, **) in columns indicate not significant at *p* > 0,05, *p* <0.05 and *p* < 0.01; respectively.

**Table 6:** Structure and information on the identified compounds in the EAF of *E. fragilis* along with individual protein ligand docking score values against bovine serum albumin (4OR0) and receptors of advanced glycated end products (4LP5).

Compounds	Informations	Chemical Structure	Docking Score (kcal/mol)	
			BSA	RAGE
Rutin	MW: 610.5 g/mol MF: C_27_H_30_O_16_ H-bound donor: 10 H-bound acceptor: 16 PubChem ID: CID 5280805	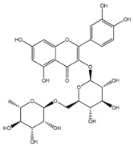	−4.41	−4.89
Caffeic acid	MW: 180.16 g/mol MF: C_9_H_8_O_4_ H-bound donor: 3 H-bound acceptor: 4 PubChem ID: CID 689043	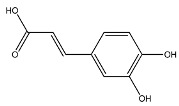	−5.84	−5.89
Ferulic acid	MW: 194.18 g/mol MF: C_10_H_10_O_4_ H-bound donor: 2 H-bound acceptor: 4 PubChem ID: CID 445858	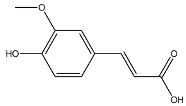	−6.35	−5.94
Galic acid	MW: 170.12 g/mol MF: C_7_H6O_5_ H-bound donor: 4 H-bound acceptor: 5 PubChem ID: CID 811292	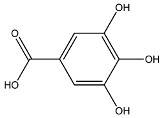	−5.25	−6.8
Quercetin	MW: 302.23 g/mol MF: C_15_H_10_O_7_ H-bound donor: 5 H-bound acceptor: 7 PubChem ID: CID 5280343	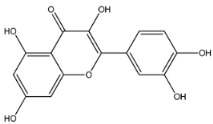	−7.7	−5.58
Vanillic acid	MW: 168.15 g/mol MF: C_8_H_8_O_4_ H-bound donor: 2 H-bound acceptor: 4 PubChem ID: CID 8468	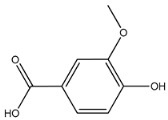	−6.05	−6.68

H-bond, hydrogen bond; MF, molecular formula; MW, molecular weight; chemical structures were retrieved from PubChem database.

## Data Availability

The data presented in this study are available on request from the corresponding author.

## Supplementary Materials

The following are available online. Supplementary File: Detailed description of the methods used for phytochemical analysis and biological activities of *E. fragilis* and its fractions.
