# Supplementary material for: Bioactive Compounds from Ephedra fragilis: Extraction Optimization, Chemical Characterization, Antioxidant and AntiGlycation Activities
Source: Molecules. 2021 Oct 2;26(19):5998. doi: 10.3390/molecules26195998 (PMC8512229; doi:10.3390/molecules26195998)
Supplement: Supplementary file 1 [file molecules-26-05998-s001.zip › molecules-1396830-supplementary.pdf]

Supplementary Materials

Phytochemical analysis

Total phenolics (TP) content

TP content was measured using the Folin-Ciocalteau reagent and gallic acid as standard as described by Slinkard and Singleton [1]. The extract samples (0.5 mL) and 2 mL of sodium carbonate (75 g. L^{-1}) were added to 2.5 mL of 10% (*v/v*) Folin-Ciocalteau reagent. After 30 min of reaction at room temperature, the absorbance was measured at 765 nm using a spectrophotometer (model UV-vis thermo Biomate 3). Tests were carried out in triplicate and results were expressed as milligrams of gallic acid equivalents per gram of dry weight of herbal mass (mg GAE/g $_{dw}$).

Total flavonoids (TF) content

TF content was determined using the aluminium chloride colorimetric method as described by Ahn et al. [2]. Briefly, 0.5 mL of 2% $AlCl_3$-ethanol solution was mixed with 0.5 mL of extract samples or standard. After 1 h at room temperature, the absorbance was measured at 420 nm. Quercetin was used as standard for the construction of calibration curve. Tests were carried out in triplicate and results were expressed as milligrams of quercetin equivalents per gram of dry weight of herbal mass (mg QE/g $_{dw}$).

In vitro antioxidant activities

DPPH radical scavenging assay

The $DPPH^{\cdot}$ free radical scavenging activity was performed according to the method of Brand-Williams et al. [3] with some modifications. Briefly, 50 µL of extract samples at different concentrations (0.1–1 mg/mL) were added to 1950 µL of 60 µM DPPH ethanol solution. The mixture was shaken vigorously and allowed to stand in the dark for 30 min at room temperature. Absorbance was recorded at 517 nm. The DPPH radical scavenging capacity (%) was calculated using the following formula:

$$\text{DPPH scavenging effect (\%)} = \left(1 - \frac{A_0}{A_1}\right) \times 100$$

where A_0 is the Absorbance of the control A_1 is the absorbance of the extract samples or standard. Ascorbic acid was used as positive control. All determinations were performed in triplicate (n = 3). The half-maximal inhibitory concentration (IC_{50}) was calculated as the concentration of extract causing a 50% inhibition of DPPH radical and expressed as the mean ± standard deviation (SD) in mg/mL.

$ABTS^{\cdot+}$ scavenging activity

$ABTS^{\cdot+}$ scavenging activity was carried out as reported by Dorman and Hiltunen [4]. ABTS and potassium persulphate were dissolved in distilled water to make the final concentration of 7 mM and 2.45 mM, respectively. $ABTS^{\cdot+}$ produced by addition of both solutions in 1:1 ratio was then incubated at room temperature for 12–16 h in the dark. The resultant intensely colored $ABTS^{\cdot+}$ solution was adjusted with ethanol to an absorbance of 0.7 ± 0.02 at 734 nm before usage. Twenty microliters of extract samples or ascorbic acid (as reference standard) at different concentrations (0.1–1 mg/mL) was added to 1980 µL of $ABTS^{\cdot+}$ solution and allowed to react for 6 min. Absorbance was measured at 734 nm against a blank (ethanol). The $ABTS^{\cdot+}$ scavenging capacity was calculated as:

$$ABTS^{\cdot+} \text{ scavenging effect (\%)} = \left(1 - \frac{A_0}{A_1}\right) \times 100$$

where A_0 is the Absorbance of the control A_1 is the absorbance of the extract samples or standard. All determinations were performed in triplicate (n = 3). The IC_{50} values were calculated and expressed as

the mean ± SD in mg/mL.

Hydrogen peroxide (H_2O_2) scavenging activity

The ability of CEE/fractions and standard (ascorbic acid) to scavenge hydrogen peroxide was determined according to the method of Ruch et al. [5]. Solution of hydrogen peroxide (40 mM) was prepared in phosphate buffer (0.1 M, pH 7.4). The extract simples or standard (1.2 mL) at different concentrations (0.1–1 mg/mL) were added to the hydrogen peroxide solution (0.6 mL,). A control was prepared containing only H_2O_2. After 10 min of incubation in the dark, the absorbance of hydrogen peroxide at 230 nm was determined against a blank solution containing phosphate buffer without hydrogen peroxide. The percentage of hydrogen peroxide scavenging of samples was calculated according to the following equation:

$$H_2O_2 \text{ Scavenging effect } (\%) = \left(1 - \frac{A_0}{A_1}\right) \times 100$$

where A_0 is the absorbance of the control, and A_1 is the absorbance in the presence of the extract samples or standard. All determinations were performed in triplicate (n = 3). The IC_{50} values were calculated and expressed as the mean ± SD in mg/mL.

Reducing power assay

The reducing power of the extract was evaluated according to Oyaizu [6]. Briefly, 0.5 mL of samples solution at different concentrations (0.1–1 mg/mL) were mixed with 2.5 mL of 0.2 M phosphate buffer (pH 6.6) and 2.5 mL of 1% (w/v) solution of potassium ferricyanide [$K_3Fe(CN)_6$]. The mixture was incubated at 50 °C for 20 min. After incubation, 2.5 mL of 10% (w/v) trichloroacetic acid (TCA) solution was added to the mixture and centrifuged at 3,000 g for 10 min. The upper layer (2.5 mL) was mixed with 2.5 mL distilled water and 0.5 mL of 0.1% (w/v) ferric chloride ($FeCl_3$). The absorbance was measured at 700 nm. Increase in absorbance of the reaction mixture indicated reducing power. The sample concentration providing 0.5 of absorbance (EC_{50}) was calculated from the graph of absorbance at 700 nm against the simple concentrations. The analyses were done in triplicate (n=3). Ascorbic acid was used as positive control.

Total antioxidant capacity (Phosphomolybdenum assay)

The assay was performed using the method applied by Trabelsi et al. [7]. Briefly, extract samples (0.1 mL) at different concentrations (0.1–1 mg/mL) were added to 1 mL of the reagent solution (containing 0.6 M sulfuric acid, 28 mM sodium phosphate and 4 mM ammonium molybdate), and the tubes were incubated at 95 °C for 90 min in a water bath. After the mixture was cooled, solution absorption was measured at 695 nm against a blank. The sample concentration providing 0.5 of absorbance (EC_{50}) was calculated from the graph of absorbance at 695 nm against the simple concentrations. The higher absorbance value indicates the greater antioxidant activity. The analyses were done in triplicate (n=3). Ascorbic acid was used as positive control.

β-Carotene blanching inhibition assay

The lipid peroxidation inhibitory of *E. fragilis* CCE/fractions was determined in β-carotene bleaching model system according to the procedure proposed by Tepe et al. [8]. In brief, 1 mL of β-carotene solution (0.5 mg/mL chloroform) was added into linoleic acid (25 μL) and 200 mg tween-40 emulsifier and vortexed for 1 min. Then the chloroform was evaporated from the mixture using a vacuum evaporator. The resulting mixture was diluted with 100 mL distilled water and vigorously agitated.

Aliquots of the reagent (2.5 mL) were then dispensed into a series of test tubes containing 350 μL extract samples or BHT (0.1 to 1 mg/mL). Immediately after reagent addition, the zero-time absorbance was measured against a blank (490 nm), which contained an emulsion without β-carotene. Next, the tubes were placed in a water bath (50 °C) to induce autoxidation, and the absorbance was recorded 120 min after incubation. β-Carotene blanching inhibition (%) was calculated using the following equation:

$$\beta - \text{Carotene blanching inhibition (\%)} = \left[1 - \left(\frac{A_0 - A_t}{A_0^{\circ} - A_t^{\circ}}\right)\right] \times 100$$

where A_0 and A_0° are the zero-time absorbance (t = 0) for test and control sample, respectively, and A_t and A_t° are the absorbance read after incubation (t = 120 min) of test and control samples, respectively. The IC_{50} values were calculated and expressed as the mean ± SD in mg/mL.

Antiglycation activity

BSA-Glucose glycation model

The inhibition of protein glycation on the BSA-Glucose model (BSA-Glu) by the CEE and its fractions was determined by the method of Adisakwattana et al. [9]. Bovine serum albumin (BSA, 10 mg/mL, in 20 mM phosphate buffer, pH 7.4, 5 mL) containing 0.1% (w/v) sodium azide was pre-incubated with extract samples dissolved in phosphate buffer (20 mM, pH 7.4, 2.5 mL) for 10 min at room temperature (20 °C). 0.5 M glucose solution (5 mL) was added to the reaction mixture. The reaction mixture without glucose was used as a blank solution. The mixtures were incubated in dark at 37 °C for 15 days. At the end of incubation period, samples were dialyzed for 24 h against the same buffer to remove unbound glucose present in solution. Samples were stored at -20 °C for further use.

Fluorometric analysis of AGEs

AGEs formation was measured using a spectrofluorometer (RF-5301, Shimazdu, Japan), with an excitation wavelength of 370 nm and an emission wavelength of 440 nm. Percent inhibition was calculated using the following Eq. (7):

$$\text{AGEs inhibition (\%)} = \left(\frac{FI_g - FI_t}{FI_g - FI_n}\right) \times 100$$

Where FI_g, FI_t and FI_n are fluorescent intensities of glycated sample, treated sample and native BSA sample respectively.

Spectroscopic analysis

The ultraviolet absorption spectra of native and glycated BSA samples were recorded in the wavelength range 250–400 nm on thermo Biomate 3 UV–vis spectrophotometer. Hyperchromicity at 280 nm was calculated using the following equation:

$$\text{Hyperchromicity (\%) at 280 nm} = \left(\frac{OD_g - OD_n}{OD_g}\right) \times 100$$

Where OD_g and OD_n are optical density at λ_{280} of glycated and native BSA sample respectively.

Determination of protein carbonyl content

The level of carbonyl group in glycated BSA was slightly modified according to Levine's method [10]. Concisely, 100 μL of glycated BSA was mixed with 400 μL of 10 mM 2,4-dinitrophenylhydrazine (DNPH) in 2.5 M HCl. After incubation for 60 min at room temperature, glycated BSA was then precipitated using 500 μL of 20% (w/v) trichloroacetic acid (TCA), left on ice for 5 min, and centrifuged at 10,000g for 10 min at 4 °C. The protein pellet was washed 3 times by 500 μL of 1:1 (*v/v*) ethanol: ethyl

acetate solution. The final protein pellet was re-suspended in 250 μL of 6 M guanidine hydrochloride. The absorbance was read at 370 nm. The protein carbonyl group of each sample was calculated by using absorption coefficient (ε = 22,000 $M^{-1}cm^{-1}$). The protein carbonyl content was expressed as nmol carbonyl/mg of protein.

Determination of thiol group

The level of thiol group in glycated BSA was determined using the Ellman's assay, with minor modifications [11]. 100 μL of glycated BSA was mixed with 300 μL 0.2 M Tris buffer (pH 8.2) and 20 μL of 0.1 mM 5,5'-Dithiobis-(2-Nitrobenzoic Acid) (DTNB). The mixture was brought to 1 mL with 0.58 mL of absolute methanol. Color was developed for 15 min and the reaction mixtures centrifuged at 3000g at room temperature for 15 min, and the absorbance was measured at 412 nm. The level of thiol group was calculated by using the extinction coefficient of the DTNB (ε = 13,600 $M^{-1}cm^{-1}$). The level of thiol group was expressed as nmol/mg of protein.

References

1. Slinkard, K.; Singleton, V.L. Total Phenol Analysis: Automation and Comparison with Manual Methods. *Am. J. Enol. Vitic.* **1977**, *28*, 49–55.
2. Ahn, M.; Kumazawa, S.; Usui, Y.; Nakamura, J.; Matsuka, M.; Zhu, F.; Nakayama, T. Antioxidant Activity and Constituents of Propolis Collected in Various Areas of China. *Food Chem.* **2007**, *101*, 1383–1392, doi:10.1016/j.foodchem.2006.03.045.
3. Brand-Williams, W.; Cuvelier, M.E.; Berset, C. Use of a Free Radical Method to Evaluate Antioxidant Activity. *LWT - Food Sci. Technol.* **1995**, *28*, 25–30, doi:10.1016/S0023-6438(95)80008-5.
4. Dorman, H.J.D.; Hiltunen, R. Fe(III) Reductive and Free Radical-Scavenging Properties of Summer Savory (Satureja Hortensis L.) Extract and Subfractions. *Food Chem.* **2004**, *88*, 193–199, doi:10.1016/j.foodchem.2003.12.039.
5. Ruch, R.J.; Cheng, S.; Klaunig, J.E. Prevention of Cytotoxicity and Inhibition of Intercellular Communication by Antioxidant Catechins Isolated from Chinese Green Tea. *Carcinogenesis* **1989**, *10*, 1003–1008, doi:10.1093/carcin/10.6.1003.
6. Oyaizu, M. Studies on Products of Browning Reaction. Antioxidative Activities of Products of Browning Reaction Prepared from Glucosamine. *Jpn. J. Nutr. Diet.* **1986**, *44*, 307–315, doi:10.5264/eiyogakuzashi.44.307.
7. Trabelsi, N.; Oueslati, S.; Falleh, H.; Waffo-Téguo, P.; Papastamoulis, Y.; Mérillon, J.-M.; Abdelly, C.; Ksouri, R. Isolation of Powerful Antioxidants from the Medicinal Halophyte Limoniastrum Guyonianum. *Food Chem.* **2012**, *135*, 1419–1424, doi:10.1016/j.foodchem.2012.05.120.
8. Tepe, B.; Daferera, D.; Sokmen, A.; Sokmen, M.; Polissiou, M. Antimicrobial and Antioxidant Activities of the Essential Oil and Various Extracts of Salvia Tomentosa Miller (Lamiaceae). *Food Chem.* **2005**, *90*, 333–340, doi:10.1016/j.foodchem.2003.09.013.
9. Adisakwattana, S.; Sompong, W.; Meeprom, A.; Ngamukote, S.; Yibchok-anun, S. Cinnamic Acid and Its Derivatives Inhibit Fructose-Mediated Protein Glycation. *Int. J. Mol. Sci.* **2012**, *13*, 1778–1789, doi:10.3390/ijms13021778.
10. Levine, R.L. Carbonyl Modified Proteins in Cellular Regulation, Aging, and Disease2,3 2Guest Editor: Earl Stadtman 3This Article Is Part of a Series of Reviews on "Oxidatively Modified Proteins in Aging and Disease." The Full List of Papers May Be Found on the Homepage of the Journal. *Free Radic. Biol. Med.* **2002**, *32*, 790–796, doi:10.1016/S0891-5849(02)00765-7.
11. Sedlak, J.; Lindsay, R.H. Estimation of Total, Protein-Bound, and Nonprotein Sulfhydryl Groups in Tissue with Ellman's Reagent. *Anal. Biochem.* **1968**, *25*, 192–205, doi:10.1016/0003-2697(68)90092-4.
